# The treatment of talar body fractures with compression screws: a case series

**DOI:** 10.4076/1757-1626-2-7953

**Published:** 2009-06-10

**Authors:** Ayhan Kilic, Yavuz Kabukcuoglu, Sami Sokucu

**Affiliations:** Department of Orthopaedic Surgery, Taksim Education and Research HospitalSiraselviler Street No: 112, Postal code: 34433, Beyoglu, IstanbulTurkey

## Abstract

Fractures of talar body are rare and serious injuries and frequently seen in multiply injured and polytraumatised patients. The high variability of talar fractures, their relatively low incidence together with the high percentage of concomitant injuries makes treatment of these injuries a challenge to the surgeon.

We treated three patients with talus body fracture and multiple articular fractures of the distal tibia. The patients were male, aged 36, 34 and 40 years. All cases were treated by open reduction and internal fixation. All the fractures were united during an average follow-up of 13 months and there were neither non-union nor collapses due to avascular necrosis.

## Introduction

Talar body fractures occur uncommonly, accounting for 7% to 38% of all fractures of the talus [1,2]. The treatment of talar body fractures remains poorly defined in the literature [1-6]. Sneppen et al. reported the early results for patients with fractures of the talar body, most of them had been treated non-operatively [7]. High rates of malunion, osteonecrosis, and arthritis were noted. There have been isolated reports of operative treatment [2-4,8-14]. The clinical outcome after talar body fractures is determined by the severity of the injury and the quality of reduction and internal fixation. However, the outcome after operative treatment remains poorly understood. The timing of definite internal fixation does not appear to affect the final result [15,16]. The incidence of avascular necrosis is almost certainly dictated by the fracture pattern and its disruption of the intrinsic blood supply to the talus [17]. The revascularisation process can be achieved by stable surgical reduction and internal fixation [5,11,14]. Thus, the importance of surgical reconstruction in displaced fractures is not only to anatomically reduce the articular surfaces and restore the dimensions of the talar body, but also to ensure that the remaining precarious blood supply to the talus is not iatrogenically reduced further [1,11,17,18].

Preoperative planning of definite internal fixation requires CT scanning. To obtain a complete intraoperative overview allowing for anatomical reconstruction of the articular surfaces and the axial deviation bilateral approaches are usually necessary. Internal fixation is achieved with screws or mini-plates supplemented by temporary K-wire transfixation in cases of marked additional ligamentous instability [18,19].

This combined injury pattern seems to be very rare; a few reports of a talar body fracture combined with ankle malleolar fractures found in the literature.

The aim of the present study was to characterize these fractures, to describe to our limited experiences about surgical treatment approach, and to evaluate the clinical, radiographic, and functional outcomes.

## Case presentation

### Case report 1

A 36-year-old male Caucasian patient who had fallen down from the third storey of a construction was admitted to the emergency room. He had swelling and haematoma over the both ankles.

**Figure 1.**
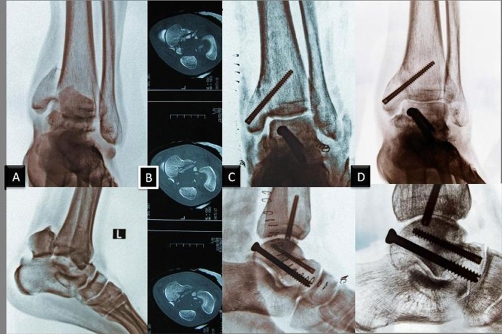
Preoperative and postoperative radiological data's of Case 1. Preoperative anteroposterior and lateral plain radiographs **(A)**. Computerized tomography scans demonstrated posterior fragment displacement with a degree of comminution of the center of the talar dome **(B)**. Postoperative anteroposterior and lateral plain radiographs; the talus body fractures and the fractures of the medial malleolus were fixed through anteromedial approach using AO cannulated and Acutrak^®^ headless screws and additional screws were inserted from posterior to anterior percutaneously. Because posteroanterior screw position gave more stability **(C)**. Anteroposterior and lateral plain radiographs following 16 months of follow-up **(D)**.

**Figure 2.**
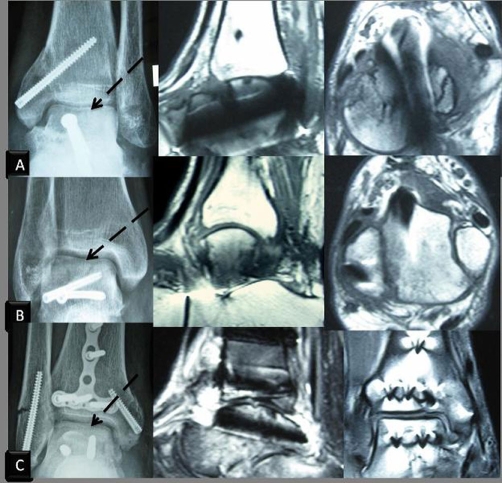
Anteroposterior radiographs (8 weeks post-op) and MRI scans (last follow-up) of patients. Anteroposterior radiographs of patients following talar body fracture, with Hawkins' sign (subchondral radiolucent band) was observed in the talar dome 8 weeks postoperatively (arrow) and MR scans of last follow-up. **(A)** Case 1; **(B)** Case 2; **(C)** Case 3.

Range of motion at the ankle, subtalar and mid-tarsal joints was painful and restricted. The tibialis posterior and dorsalis pedis arteries were detected with Doppler ultrasound. Radiographic examination revealed a tibia plafond fracture, a distal fibula fracture and a talo-tibial divergent fracture-dislocation in the right lower extremity; while in the left lower extremity there was a fracture of the medial malleolus and an 81C3 talus body fracture according to the OTA classification. Soft tissue injury was assessed (Tscherne classification-G1) while CT was demonstrated to posterior fragment displacement with a degree of comminution of the center of the talar dome. It's essential for exact assessment and preoperative planning. The patient was hospitalized after applied below knee plaster cast for the control of edema. In the surgical intervention performed under spinal anaesthesia in the second day following the accident, circular fixation was used for the tibia plafond fracture and the distal fibula fracture was plated in the right lower extremity. The talus body fractures and the fractures of the medial malleolus in the left lower extremity were fixed through anteromedial approach using 4. 5 mm AO cannulated and headless screws (Acutrak^®^ Acumed, Hillsboro, OR). The patient was discharged after three days. Ankle movement without weight-bearing was allowed for a period of three months. The fractures of the talus and the medial malleolus united at the end of sixth week. The circular fixator on the right lower extremity was taken off in the second month following the operation and movements were allowed. At 16 months follow-up, the patient had dorsiflexion of 15°, plantar flexion of 20° and full range of subtalar joint movements. He had a stable ankle and minimal pain on walking on uneven ground. The radiographs taken at this time showed no evidence of avascular necrosis. Clinical results were evaluated using the clinical rating scale of the American Orthopaedic Foot and Ankle Society (AOFAS). His score was 86 points (Figures 1, 2).

### Case report 2

**Figure 3.**
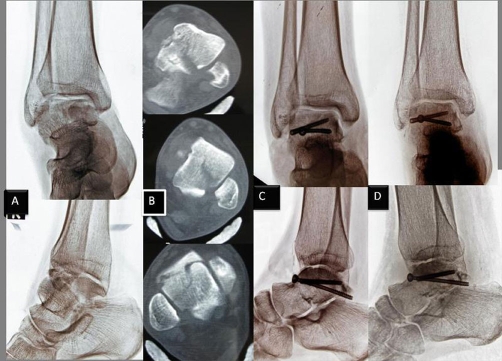
Preoperative and postoperative radiological datas of case 2. Preoperative anteroposterior and lateral plain radiographs **(A)**. CT scans demonstrated a talar body fracture lines associated with comminution **(B)**. The patient underwent open reduction and internal fixation through anterolateral approaches **(C)**. 12 months later, the fracture had united with a congruent ankle joint and without evidence of osteonecrosis **(D)**.

A 47-year-old Caucasian male driver was injured in a head-on collision while driving a car. On arrival at the hospital, fractures were identified at the left femoral shaft, subtrochanteric and intertrochanteric regions, the left tibial plateau (Gustilo-Anderson type I open fracture), a rib in the left chest, the left proximal humerus and the right talus body (OTA class. 81C3). The patient was operated under general anaesthesia in the second day following the accident. The femoral shaft and intertrochanteric fractures was fixed with a reconstruction nail while the proximal humerus fracture was fixed with a locking plate (Polarus^®^Acumed, Hillsboro, OR). The other fractures were fixed using multiple AO cannulated and headless compression screw (Acutrak^®^). Anterolateral approach was used for the talar body fracture. The patient was discharged after ten days. Ankle movement without weigh-bearing was allowed for a period of three months. The fracture of the talus united at the end of eight weeks. At five months, the other fractures had healed completely. At 12 months follow-up, the patient had dorsiflexion of 10°, plantar flexion of 20° and full range of subtalar joint movements. The patient had some pain while walking however, ankle and subtalar movements were normal. His AOFAS score was 78 points (Figures 2, 3).

### Case report 3

**Figure 4.**
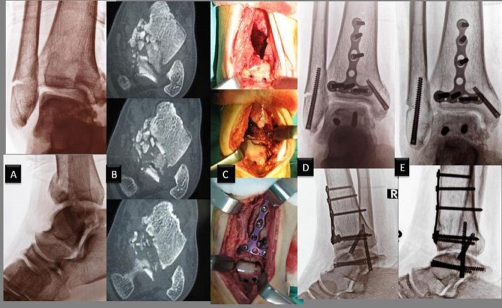
Radiological and intra-operative data's of Case 3. Anteroposterior and lateral radiographs of the ankle of a 33-year-old male who had sustained a comminuted talar body fracture as the result of a fall from a height. Fractures were identified at the right tibia pilon (AO/OTA type C1), lateral malleolar fracture and talus body (OTA class. 81 C3) **(A)**. CT scanning revealed severe comminution of the posterior part of the body **(B)**. Intraoperative photographs demonstrate an anterior approach the fractures are seen before and after the reduction. The fractures were fixed with multiple headless compression screw and locking plate system for distal tibia **(C)**. Postoperative anteroposterior and lateral plain radiographs **(D)**. 12 months later, the fracture had united with a minimal degenerative changes in ankle joint and without evidence of osteonecrosis **(E)**.

A 33-year-old Caucasian male had fallen off from a height 3 m. Fractures were identified at the right tibia pilon (AO/OTA type C1) and talus body (OTA class. 81 C3) on arrival at the hospital. The patient was operated under spinal anaesthesia on the second day following the accident. The fractures were fixed with multiple headless compression screw and locking plate system for distal tibia (Acumed, Hillsboro, OR). Anterior approach was used for internal fixation. The patient was discharged on the fourth day following the operation. The fracture of the talus united at the end of eight weeks. At 12 months follow up, the patient had dorsiflexion of 5°, plantar flexion of 10° and full range of subtalar joint. He had a stable ankle and no pain on walking on uneven ground. His AOFAS score was 87 points (Figures 2, 4).

## Discussion

Talar body fractures are extremely rarer still. The body of the talus articulates with tibia, fibula and the calcaneus. Complexity in the blood supply to the talus itself makes it one of the bones in the body vulnerable to avascular necrosis [17]. Arthritis in the ankle and subtalar joints can occur in the absence of avascular necrosis of the talus and joint incongruity [6,16].

The reported incidence of avascular necrosis for severely comminuted talar body fracture is around 50%-75% [5,10,12,20-22]. *Vallier et al.* reporting on radiographic findings of 26 talar body fractures with a minimum follow-up of 1 year, noted a 38% incidence of AVN, 65% incidence of post-traumatic tibiotalar arthritis and 34% incidence of posttraumatic subtalar arthritis. Worse outcomes were noted in association with comminuted fractures, associated talar neck fractures and open fractures [4]. *Lindvall et al.*, in 2004, reported on 26 isolated cases of talar neck and body fractures with a minimum follow-up of 48 months and found a 50% incidence of AVN and 100% incidence of post-traumatic arthritis. Timing of fixation did not seem to affect the outcome, union or prevalence of AVN in the later study [10].

The appearance of a radiolucent zone 4-8 weeks after the injury at the subcortical bone of the talar dome indicating bone remodelling “Hawkins' sign” is highly predictive of a revitalisation of the talar body after a fracture. We had not seen AVN in our patients. All cases with a positive Hawkins sign. Thirteen months (mean follow-up period) after the initial injury, our patient made full recovery with no evidence of avascular necrosis or collapse radiologically and clinically. The reason for not seeing avascular necrosis, however, could be our short follow-up period. The mild osteo-arthritic changes seen at the 3 years follow-up are expected in our patient, since the incidence of degenerative changes after talar body fractures is high, affecting more often the tibiotalar than the subtalar joint.

Talar body fractures are produced by an axial compression of the talus between the tibial plafond and calcaneus [6,12]. In cases with a combined medial malleolar fracture, an additional inversion torque seems to distribute this force to the medial structures, producing a vertical split of the talar body and the medial malleolar fracture [8]. A lateral side talar body fracture could be produced by pronation-external rotation [7]. Recently, *Frawley et al.* showed in their series of 26 talar fracture patients, 15 talar fractures in the right feet, which push brake pedals, in 16 car drivers [23]. Fractures in the neck and the lateral process of the talus were believed to occur when both the foot and ankle are hyperdorsiflexed [9]. The fracture pattern in our cases suggests an inversion, axial loading, and external rotation mechanism.

In our patients, open reduction and internal fixation was performed with the use of 4.5 mm AO cannulated or headless cannulated (acutrak^®^, Acumed) screws. We performed surgery through three different (single) approach individually (case1, anteromedial; case 2, anterolateral; case 3, anterior approach). The screws are introduced into the posterior part of the talar body in order to achieve maximum purchase. On the other hand, additional screw was inserted percutaneously. Because posteroanterior screw position gave more stability in a biomechanical trial [19].

Open reduction and internal fixation may restore the joint congruity allowing early range of movement in the tibio-talar and subtalar joints. Plaster cast application was not used and range of motion exercise was encouraged at the early post-operative period but full weight bearing was allowed at 12 weeks [8].

## Conclusion

Fractures of the talar body are often severe injuries. Conservative treatment with closed reduction and casting leads to a very high rate of complications. Hence, open reduction and internal fixation in the appropriately selected patient can be performed safely with the prospect of reducing complications. An accurate reduction and stable fixation are also mandatory in order to provide the best biomechanical environment for revascularization of the lateral part of the talar body. Early treatment with open reduction and stable internal fixation, using minimal invasive technique, may improve the final outcome.

## List of abbreviations

None.
